# Stretch-Sensitive Down-Regulation of the miR-144/451 Cluster in Vascular Smooth Muscle and Its Role in AMP-Activated Protein Kinase Signaling

**DOI:** 10.1371/journal.pone.0065135

**Published:** 2013-05-21

**Authors:** Karolina M. Turczyńska, Anirban Bhattachariya, Johanna Säll, Olga Göransson, Karl Swärd, Per Hellstrand, Sebastian Albinsson

**Affiliations:** Department of Experimental Medical Science, Lund University, Lund, Sweden; Virginia Commonwealth University, United States of America

## Abstract

Vascular smooth muscle cells are constantly exposed to mechanical force by the blood pressure, which is thought to regulate smooth muscle growth, differentiation and contractile function. We have previously shown that the expression of microRNAs (miRNAs), small non-coding RNAs, is essential for regulation of smooth muscle phenotype including stretch-dependent contractile differentiation. In this study, we have investigated the effect of mechanical stretch on miRNA expression and the role of stretch-sensitive miRNAs for intracellular signaling in smooth muscle. MiRNA array analysis, comparing miRNA levels in stretched versus non-stretched portal veins, revealed a dramatic decrease in the miR-144/451 cluster level. Because this miRNA cluster is predicted to target AMPK pathway components, we next examined activation of this pathway. Diminished miR-144/451 expression was inversely correlated with increased phosphorylation of AMPKα at Thr172 in stretched portal vein. Similar to the effect of stretch, contractile differentiation could be induced in non-stretched portal veins by the AMPK activator, AICAR. Transfection with miR-144/451 mimics reduced the protein expression level of mediators in the AMPK pathway including MO25α, AMPK and ACC. This effect also decreased AICAR-induced activation of the AMPK signaling pathway. In conclusion, our results suggest that stretch-induced activation of AMPK in vascular smooth muscle is in part regulated by reduced levels of miR-144/451 and that this effect may play a role in promoting contractile differentiation of smooth muscle cells.

## Introduction

Vascular smooth muscle cells (VSMCs) have a remarkable ability to adapt to environmental cues by phenotypic modulation [1]. This is likely a key mechanism to allow for repair following vascular injury but phenotypic modulation of smooth muscle cells can also have detrimental effects in the development of vascular disease [2]. Intense efforts have therefore been made to clarify the molecular mechanisms behind phenotypic modulation of VSMCs and to identify potential targets for therapeutic intervention in this response.

We have previously reported that mechanical stretch is an important factor for the regulation of smooth muscle phenotype using murine portal veins in organ culture [3]–[7]. In this model, portal veins are subjected to longitudinal stretch by applying a gold weight at one end of the vessel, which results in increased hypertrophic growth, remodeling and contractile differentiation over one to five days. Although multiple pathways are likely to be involved in smooth muscle mechanotransduction, stretch partly promotes growth by activating MAP-kinase/ERK and PI3-kinase/Akt, and contractile differentiation by stimulating the Rho/cofilin pathway and actin polymerization. These signaling pathways are also known to be activated in the arterial system during experimental models of hypertension [8]–[14]. Stretch-induced growth in the portal vein is also dependent on endogenous release of angiotensin II and endothelin-1 [3], [15], while contractile differentiation requires calcium influx via L-type calcium channels [16], [17].

MicroRNAs are short, non-coding RNAs that are known to regulate mRNA stability and protein translation by binding to their target mRNAs [18]. In most cases, miRNA-binding to the 3′UTR of the target mRNA results in translational inhibition or mRNA degradation. In smooth muscle, the role of miRNAs has been investigated using conditional knockout of Dicer, an essential enzyme for miRNA maturation. Early embryonic deletion of *Dicer* in smooth muscle causes embryonic lethality due to reduced proliferation, contractile differentiation and contractile function of smooth muscle cells, which results in widespread internal hemorrhage [19]. Similar effects on smooth muscle differentiation and contractile function are observed in adult mice with inducible and smooth muscle specific deletion of *Dicer* [20], [21]. In a recent report, we found that inducible deletion of Dicer in smooth muscle results in loss of L-type calcium channels and reduced stretch-induced contractile differentiation of the portal vein [17].

In the present study we hypothesized that miRNAs are regulated by mechanical factors and play a role in stretch-induced signaling events in vascular smooth muscle cells. We used portal veins in organ culture to screen for miRNAs that are regulated by mechanical stretch and found a cluster of two miRNAs, miR-144/451 that were significantly down-regulated and may play a role in stretch-dependent activation of the AMP-activated protein kinase (AMPK) pathway and contractile differentiation.

## Materials and Methods

### Ethics statement

All animal work was conducted according to national and international guidelines and approved by The Malmö/Lund ethical committee on animal experiments (M260-11).

### Chemicals

AICAR (AMPK activator) was purchased from Toronto Research Chemicals (TRC), #A611700 and dissolved in water to 250 mM by warming to 37°C and vortexing.

### Organ culture

Adult male C57BL/6J mice (30–35 g) were euthanized by cervical dislocation. Portal veins were freed from fat and surrounding tissue and mounted on a hook in a test tube containing DMEM/Hams F12 with 2% dialyzed FCS and 10 nM insulin as described [4]. All of the blood was carefully removed from the vessels, which were either stretched by attaching a 0.3 g gold weight at one end or left non-stretched as controls. The load corresponds to the optimal load for force development. The vessels were incubated in cell culture environment from 24 hours up to 5 days.

Carotid arteries were mounted on glass cannulas in a pressure myograph chamber (Living Systems Instrumentation, Burlington, VT) and secured with silk sutures as described previously [22]. The vessels were then incubated under zero or 95 mmHg intraluminal pressure in HEPES buffer (composed of 135.5 mM NaCl, 5.9 mM KCl, 2.5 mM CaCl_2_, 1.2 mM MgCl_2_, 11.6 mM glucose, and 11.6 mM HEPES, pH 7.4). The vessel diameter was monitored using a Nikon Diaphot 200 inverted microscope equipped with a charge-coupled device (CCD) camera and VediView 1.2 software (Danish MyoTechnology). The intraluminal pressure was continuously monitored by two pressure transducers (one on the inflow side other on the outflow side), which were connected to a pressure servo and peristaltic pumps (Living Systems Instrumentation, Burlington, VT). The temperature was maintained at 37°C throughout the experiment.

### Cell culture and transfection

Vascular smooth muscle cells were isolated from mouse aorta by enzymatic digestion as described previously [19]. Mouse aortic smooth muscle cells (mAoSMCs) were used at passages 3–5 for experiments. Cells were transfected with commercially available synthetic microRNA mimics for miR-144, miR-451 (Syn-mmu-miR-144, Syn-mmu-miR-451 Qiagen or MISSION^®^ microRNA-144 or microRNA-451 mimic, Sigma) or negative control (MISSION^®^ microRNA negative control, Sigma) using Oligofectamine transfection reagent (Invitrogen) as described previously [19].

### MicroRNA array

Mouse portal veins were organ cultured for 24 hours either stretched our unstretched (n = 14). The portal veins were then pooled and the miRNA-enriched fraction was isolated using miRNeasy mini kit (Qiagen, #217004) and RNeasy MinElute Cleanup Kit (Qiagen, #74204) according to the manufacturer's instructions. Following reverse transcription using RT^2^ miRNA First Strand Kit (SA Biosciences #331401), the expression of 528 miRNAs was analyzed using RT^2^ miRNA PCR Array mouse (SA Biosciences #MAM-200C).

### Quantitative real-time PCR (qRT-PCR)

Total RNA was isolated using miRNeasy mini kit (Qiagen, #217004) according to the manufacturer's instructions. For miRNA detection 250–500 ng of template RNA was reverse transcribed to cDNA using miScript II RT Kit (Qiagen, #218161) according to the manufacturer's instructions. The relative expression of miRNAs was analyzed by real-time qPCR (StepOnePlus qPCR cycler, Applied Biosystems) using miScript SYBR Green PCR Kit (Qiagen, # 218073) and miScript Primer Assays (Mm_miR-144_3, #MS00032326; Mm_miR-451_1 # MS00002408, Hs_RNU6-2_1, #MS00033740).

For mRNA analysis, the same isolation kit, including on-column DNAse I digestion, and qPCR equipment as for miRNA was used, but mRNA expression was detected using QuantiFast SYBR Green RT-PCR Kit Qiagen, #204156) and QuantiTect primer assays (Qiagen, Mm_Kcnmb1, #QT00101500, Mm_Tagln, QT00165179, Mm_Cnn1, QT00105420, Mm_Des, #QT00102333). The qPCR primer sequences used for Mm_Myh11 have been published previously [23].

### Western blotting

Cells grown on 6-well plates were washed twice with ice-cold PBS and lysed on ice directly in the wells with 70–75 µl of 1× Laemmli sample buffer (60 mM Tris-HCl, pH 6.8, 2% SDS, 10% glycerol). After protein determination with the Lowry method (BioRad reagents) bromophenol blue and β-mercaptoethanol were added to the samples at final concentrations of 0.005% and 5% respectively. Equal amounts (15–30 µg) of protein were loaded in each lane of Bio-Rad TGX 4–15% Criterion gels. Proteins were then transferred using either wet transfer over-night or semi-dry transfer for 10 min using the Trans-Blot Turbo system (Bio-Rad). Proteins were detected using commercially available primary antibodies: AMPKα (#2603), ACC (#3662), MO25α (CAB39, #2716), p70 S6 Kinase (#9202) (1∶1000), T172-phospho-AMPKα (#2535), S79-phospho-ACC (#3661), T389-phospho-p70 S6 Kinase (#9234) (1∶500) - Cell Signaling, GAPDH (#MAB374, Millipore, 1∶1000–1∶5000), HSP90 (#610418, BD Transduction Laboratories, 1∶1000). Secondary mouse or rabbit HRP-conjugated antibodies (#7074, #7076 1∶5000 or 1∶10000, Cell Signaling) were used. Bands were visualized using ECL (Pierce West Femto) and images were acquired using the Odyssey Fc Imager (LI- COR Biosciences).

### AMPK activity assay

Cell lysates containing 5–25 µg total protein were subjected to immunoprecipitation of AMPKα1 and -α2 with 1 µg of isoform-specific antibodies, kindly provided by Professor Grahame Hardie, University of Dundee. Kinase activity against a peptide substrate was then determined in vitro as described previously [24]. In short, the phosphotransferase activity was measured by incubating the immunoprecipitates for 20 min at 30°C, in a total assay volume of 50 µl, containing 50 mM Tris-HCl pH 7.5, 0.1% (v/v) 2-mercaptoethanol, 10 mM MgCl_2_, 0.1 mM EGTA, 0.1 mM [γ-^32^P]-ATP (300 cpm/pmol) (PerkinElmer) and 200 µM of the AMARA (AMARAASAAALARRR) peptide substrate (GL Biochem Ltd). The reaction mixture was applied onto P81 papers (Whatman), which were washed with 50 mM phosphoric acid, and the incorporated radioactivity was then quantified by scintillation counting.

### Statistics

Values are presented as mean ± S.E. unless otherwise stated. P-values were calculated by Student's t-test or one-way analysis of variance followed by Bonferroni post-hoc testing using GraphPad Prism 5 (GraphPad Software Inc.). P<0.05 was considered statistically significant. Data are expressed as mean ± S.E.M. *, p<0.05; **, p<0.01; ***, p<0.001.

## Results

### The expression of microRNA-144/451 is down-regulated in stretched portal veins and in pressurized carotid arteries, which inversely correlates with increased expression and phosphorylation of AMPK

**Figure 1:**
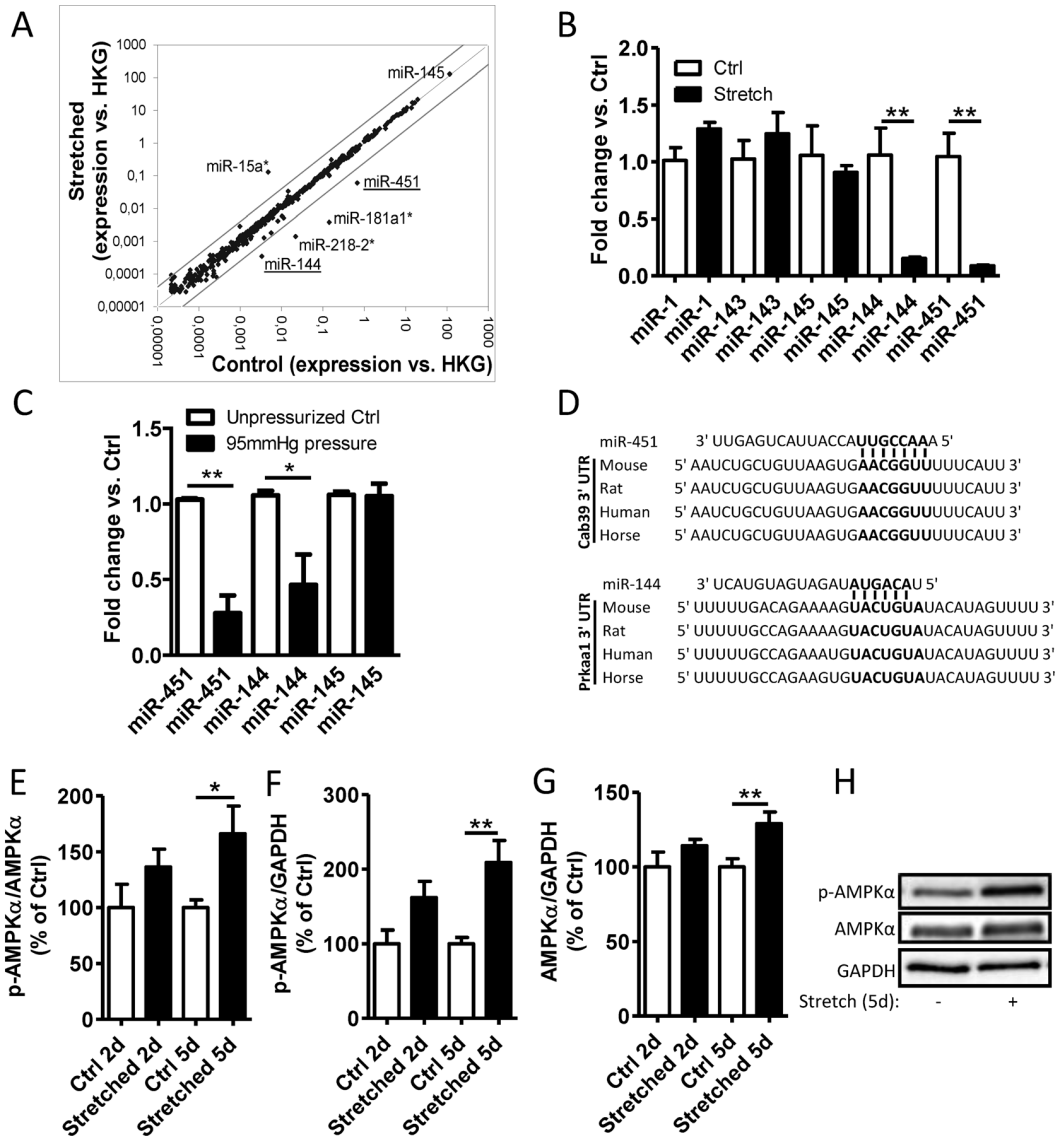
Stretch-sensitive miRNA expression and AMPK activation in stretched portal veins. (A) Scatter plot of miRNAs expression levels analyzed by qPCR based miRNA array in stretched versus non-stretched (control) portal vein after 24 hours organ culture. HKG refers to four different house-keeping genes used for data normalization and miRNA* represents the passenger strand of mature miRNAs. Grey lines indicate 4-fold up- and down-regulation, respectively. (B) Confirmation of the array analysis by individual qPCR reactions in triplicate. (C) Mouse carotid arteries were incubated for 6 hours *ex vivo* with or without intraluminal pressure (95 mmHg). MicroRNAs were then analyzed by qPCR. (D) Predicted target site for miR-451 in the 3′ UTR of MO25α (*Cab39*) mRNA and for miR-144 in the 3′UTR of AMPKα1 (*Prkaa1*) mRNA. (E–H) Activation of AMPK signaling was analyzed by western blotting and phospho-specific antibodies in portal veins stretched for 2 days (2 d) or 5 days (5 d) (n = 4–10). The phosphorylation level of AMPKα at T172 was compared to either total AMPKα (E) or GAPDH (F). Total content of AMPKα versus GAPDH is shown in (G). (H) Representative western blot of T172-phospho- and total-AMPKα and GAPDH in portal veins stretched for 5 days.

To identify miRNAs sensitive to stretch, we performed whole genome miRNA qPCR arrays using RNA extracted from pooled mouse portal veins (n = 14) stretched for 24 h or unstretched controls and normalized to four different housekeeping genes (HKG). The analysis revealed a dramatic decrease in miR-144 and miR-451 with only minor changes (4-fold cut off) in other functional miRNAs strands including the smooth muscle specific miR-145 (Figure 1A). The down-regulation of miR-144 and miR-451 in stretched portal veins was then validated using individual qPCR reactions in triplicate (Figure 1B). Some of the miRNAs that were not affected by stretch at 24 h such as miR-1, miR-143 and miR-145 were also included in the individual miRNA analysis (Figure 1B). In accordance with the effect in portal veins, pressurization of carotid arteries for 6 hours resulted in decreased expression of miR-144/451 (Figure 1C).

An analysis of predicted targets of miR-451 and miR-144 revealed MO25α (*Cab39*) and AMPKα1 (*Prkaa1*), respectively (Figure 1D). Both MO25α and AMPKα are involved in the regulation of the AMPK pathway, a central regulator of cellular metabolism. Furthermore, AMPK has been demonstrated to be activated by AngII [25] and to play an important role in prevention of neointimal hyperplasia [26], [27].

In order to investigate the effect of stretch on AMPK signaling we analyzed phosphorylation of AMPKα in stretched versus non-stretched portal veins. Phosphorylation at Thr172, which governs AMPK activity, was significantly increased in portal veins stretched for 5 days both when compared to total AMPKα expression levels (Figure 1E and H) and to GAPDH (Figure 1F and H). Total AMPKα protein content was increased after 5 days of stretch (Figure 1G and H), inversely correlating with the down-regulated levels of miR-144 and miR-451 in these vessels. No significant change in either AMPK phosphorylation or AMPK protein content was detected in stretched portal veins after 2 days of organ culture.

### Transfection of miR-144/451 mimics leads to a down-regulation of AMPKα/MO25α/ACC protein expression in primary VSMCs

To evaluate the effect of miR-144 and miR-451 on the expression of mediators in the AMPK pathway, we transfected primary VSMCs isolated from mouse aorta with miRNA mimics either separately or in combination. The expression levels of both miRNAs were very low in cultured cells compared to intact smooth muscle (Suppl. Figure S1A and B) and therefore we did not attempt to inhibit these miRNAs using miRNA inhibitors. qPCR analysis confirmed that the miR-144 or miR-451 expression level in cells transfected with the respective miRNA mimic was significantly higher than in the cells transfected with negative control (data not shown). Both mimics were used at 100 nM concentration, which was chosen based on dose-response experiments (data not shown).

**Figure 2:**
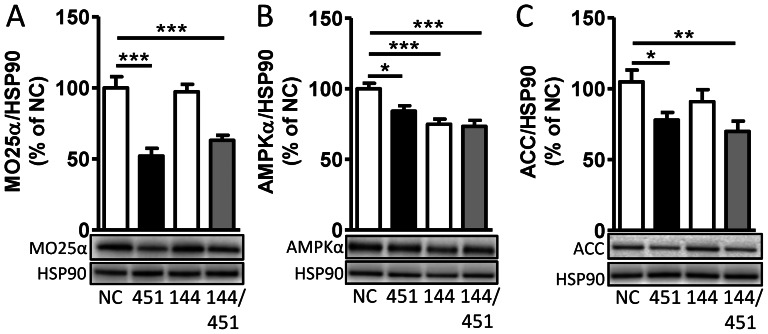
The miR-144/451 cluster reduces the protein levels of MO25α, AMPKα and ACC. Primary mouse aortic smooth muscle cells were transfected with synthetic mature mimics of either miR-144 (100 nM), miR-451 (100 nM) or miR-144+miR-451 (50 nM each). After 96 hours, the cell lysates were immunoblotted with anti-MO25α/CAB39 (A), anti-AMPKα (B) or anti-ACC (C). The graphs show quantification of 3 independent experiments in duplicates normalized to cells transfected with negative control (NC). HSP90 was used as loading control. A representative blot is shown below each graph.

MO25α, which is a scaffolding protein required for full activity of the upstream AMPK kinase - LKB1, has previously been experimentally validated as a target of miR-451 in glioma cells [28] and cardiac myocytes [29]. In accordance with these studies we found a down-regulation of this protein following transfection of miR-451 mimic in primary VSMCs (Figure 2A). Furthermore, transfection of either miR-144 or miR-451 mimic down-regulated the protein expression of AMPKα (Figure 2B). Unexpectedly transfection with miR-451 also affected the protein expression of acetyl-CoA carboxylase (ACC), which is a downstream mediator of the AMPK pathway.

### Transfection of miR-451 and miR-144 mimics reduces AICAR- induced activation of the AMPK pathway

In order to evaluate the importance of miR-144 and miR-451 for the activity of AMPK signaling, smooth muscle cells were transfected with miRNA mimics and analyzed by western blot using phospho-specific antibodies. Since total levels of AMPK and ACC are reduced by the miR-144 and miR-451 mimics, phosphorylated AMPK and ACC levels were normalized to the stably expressed heat-shock protein 90 (HSP90) to reveal the full effect of the miRNA mimics.

**Figure 3:**
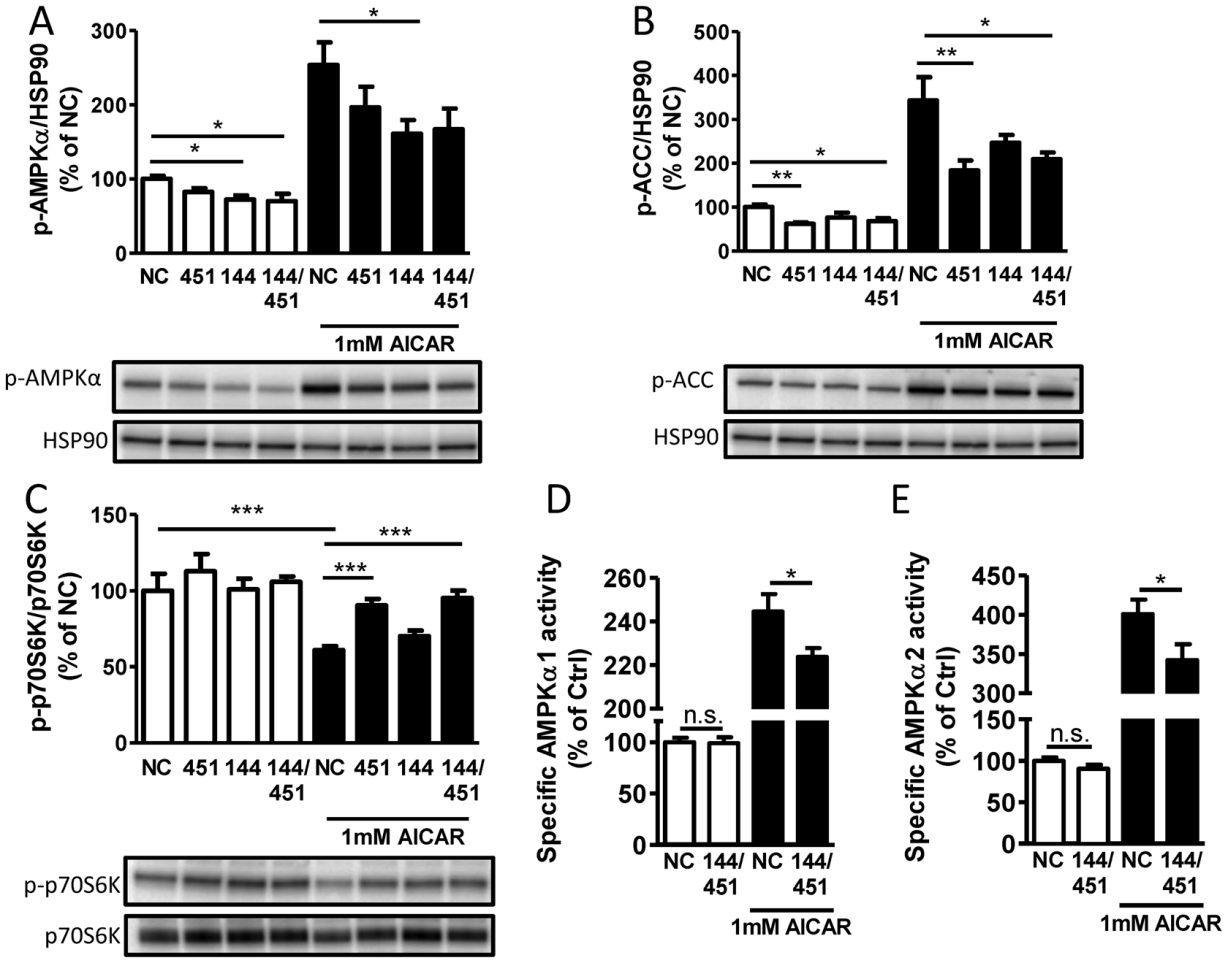
Effect of miR-144/451 mimic on basal and AICAR-induced AMPK signaling. Primary mouse aortic smooth muscle cells were transfected with synthetic mature mimics of either miR-144 (100 nM), miR-451 (100 nM) or miR-144+miR-451 (50 nM each). After 72 hours the cells were starved in 0.1% FBS DMEM/Ham's F12 media for 24 hours and then treated with 1 mM AICAR for 20 minutes. Total cell lysates were immunoblotted for T172-phospho-AMPK (A), S79-phospho-ACC (B) and T389-phospho-p70S6K (C) or analyzed by *in vitro* AMPK activity assay using AMARA as a peptide substrate (D and E). For the AMPK activity assay, absolute values in control samples were 409–449 mU/mg protein for AMPKα1 and 9.6–14.4 mU/mg protein for AMPKα2, where 1 mU represents pmol/min of incorporated ATP. All graphs show quantification of 2–3 independent experiments in duplicates or triplicates normalized to untreated cells transfected with negative control (NC). HSP90 or total p70S6K was used as loading control. A representative blot is shown.

After 72 hours of transfection, the cells were starved for 24 hours and then treated with the AMPK activator AICAR (1 mM) for 20 min. As expected, AICAR caused a significant increase of both AMPK phosphorylation at Thr172 and phosphorylation of its downstream target ACC at Ser79. Notably, the increase in AMPK phosphorylation by 1 mM AICAR was approximately 150% (Figure 3A), which is in the same range as the increase caused by mechanical stretch (Figure 1F). Although miR-144 mimic was most effective in inhibiting AMPK phosphorylation, miR-451 mimic also prevented part of the AICAR-induced response (Figure 3A). As shown in Figure 3B, phosphorylation of ACC was reduced by miR-451 mimic in both basal conditions and after stimulation with AICAR, while miR-144 had a slightly smaller effect.

The activity of mTOR signaling, which is negatively regulated by AMPK, was measured by phosphorylation of its downstream target p70 ribosomal S6 kinase (p70S6K) at Thr389. As expected, AICAR treatment reduced p70S6K phosphorylation and we found that transfection of miR-451 mimic alleviated this inhibition (Figure 3C).

To test if the effect of miR-144/451 on AMPK phosphorylation correlated with an effect on AMPK activity, cells were treated with the combination of miR-144/451 according to the same protocol as in Figure 3A–C and analyzed by *in vitro* AMPK activity assay. As shown in Figure 3D and E, transfection of miR-144/451 mimic resulted in a small but significant decrease in AICAR-induced AMPKα1 and AMPKα2 activity.

### AICAR-induced AMPK activation promotes contractile differentiation of vascular smooth muscle

**Figure 4:**
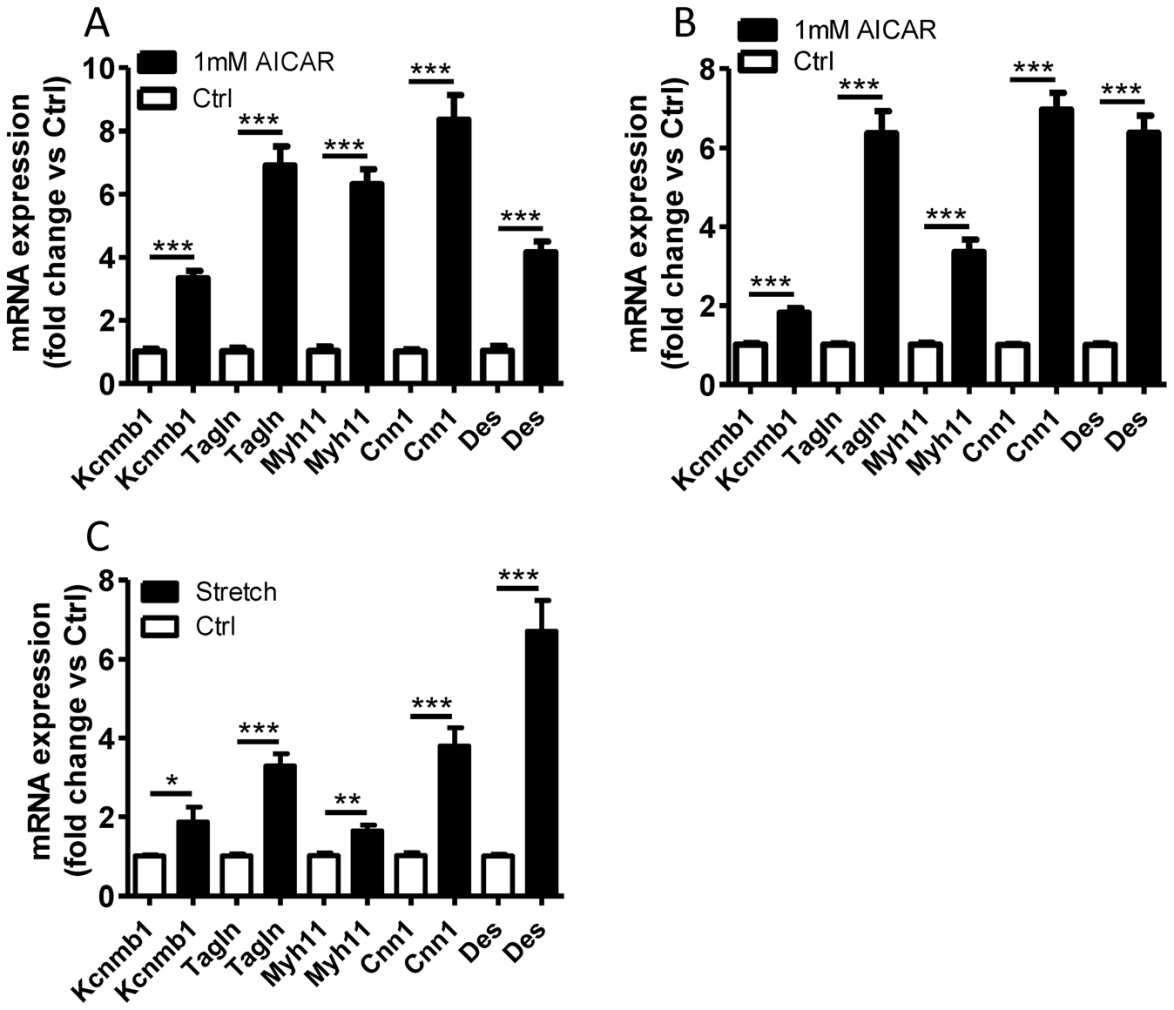
AICAR treatment of either aorta or unstretched portal vein increases mRNA expression of smooth muscle markers and mimics the effect of stretch. Aorta (A) and unstretched portal veins (B) were treated with 1 mM AICAR or stretched (C) for 24 hours and then analyzed for calponin (*Cnn1*), BKchannel β_1_-subunit (*Kcnmb1*), desmin (*Des*), myosin heavy chain (*Myh11*) and SM22 (*Tagln*) expression by qPCR (n = 4).

Activation of AMPK has previously been implicated in the regulation of smooth muscle contractile differentiation [30] and it is well known that mechanical stretch of the vascular wall promotes the expression of smooth muscle markers [16], [17], [31]. In order to test if the level of AMPK-activation induced by mechanical stretch is sufficient to promote contractile differentiation, we stimulated isolated intact aorta (Figure 4A) and unstretched portal veins (Figure 4B) with 1 mM AICAR for 24 h in organ culture. The mRNA expression of known stretch-sensitive smooth muscle markers such as calponin (*Cnn1*), desmin (*Des*), SM22 (*Tagln*), the BK channel β_1_-subunit (*Kcnmb1*) and myosin heavy chain (*Myh11*) was then analyzed by qPCR. AICAR promoted the expression of these smooth muscle markers to a similar or slightly higher extent compared to mechanical stretch of the portal vein (compare with Figure 4C).

## Discussion

In this study we investigated how the miRNA expression profile changes in response to stretch and tested the effect of stretch-sensitive miRNAs on intracellular signaling in vascular smooth muscle. We show that miR-144/451 expression is significantly and specifically reduced in stretched portal veins and pressurized carotid arteries. Furthermore, we demonstrated that over-expression of these miRNAs affects the regulation of AMPK signaling by down-regulating total protein levels of MO25α, AMPKα and ACC. This effect resulted in inhibition of AICAR-induced phosphorylation and activation of AMPK and prevention of AICAR-induced inhibition of ACC and p70S6K. Finally, we demonstrate that AICAR can promote contractile differentiation in non-stretched portal vein, similar to the effect of mechanical stretch.

We have previously demonstrated that miRNAs are essential for stretch-induced contractile differentiation, partly via reduction of L-type channel expression, which is likely due to increased expression of CamKIIä, a known target of miR-145 [17]. However, in that study we did not investigate the effect of stretch on miRNA expression and the role of stretch-induced miRNAs in intracellular signaling events in vascular smooth muscle. A number of specific miRNAs including miR-21 and miR-26a, and miR-153/223 have previously been demonstrated to be regulated by stretch in isolated and cultured vascular [32], [33] and airway smooth muscle cells [34], respectively. Collectively, these miRNAs were shown to promote proliferation or hypertrophic growth of smooth muscle cells. Although studies in isolated cells are important to define the molecular mechanisms of stretch-induced signaling it is well known that tissue composition and contractile differentiation are essential factors for the mechanical properties of vascular smooth muscle. Therefore, stretch-induced effects also need to be investigated in systems in which the contractile phenotype and cell-matrix interactions are maintained. To our knowledge this is the first study investigating the effects of pressure and stretch on miRNA expression in intact vessels.

Since the turnover of miRNAs in quiescent smooth muscle is relatively slow, the effects on miR-144/451 observed in this study should be considered an early event in stretch-induced miRNA expression. MiR-144 and/or miR-451 have been studied previously in numerous cell types including erythrocytes [35], [36], glioma cells [28], cardiomyocytes [29], [37], [38] and smooth muscle cells [39]. In both glioma cells [28] and cardiomyocytes [29], miR-451 has been demonstrated to target MO25α, an important regulator of the AMPK-signaling pathway [40]. We could confirm this effect in smooth muscle and also demonstrate that both miR-144 and miR-451 directly or indirectly target other mediators of the AMPK pathway including AMPK itself, and ACC.

AMPK activity is also regulated by phosphatases and miR-144 is predicted to target regulatory subunits of both protein phosphatase 1 (PP1) and protein phosphatase 2 (PP2). Although we did not investigate the role of PP1 and PP2 in the present study, we predict that it is unlikely that the inhibitory effect of miR-144 on AMPK activity is due to induction or activation of these phosphatases. In contrast, a reduced expression of the regulatory subunits of PP1 and PP2 by miR-144 would be expected to augment AMPK phosphorylation [41].

To our knowledge, stretch-induced activation of AMPK in smooth muscle has not been demonstrated previously and thus represents a novel mechanism for smooth muscle mechanotransduction. Recent reports have shown that AMPK can be activated by Ang II [25] and inhibit the proliferative response to AngII in vascular smooth muscle cells. One can speculate that the activation of AMPK by stretch could act as a negative feedback mechanism to prevent excessive growth in response to stretch. This may have important clinical implications since deletion of AMPK exacerbates neointimal hyperplasia following vascular injury [26] while activation of AMPK by systemic or local delivery of AMPK activators like AICAR or A 769662, results in a significant reduction in neointimal area [27]. Future studies are needed in order to investigate if inhibition of miR144/451 in vascular smooth muscle cells can reduce neointimal hyperplasia via potentiation of AMPK signaling.

Although AMPK is mostly recognized for its anti-proliferative effect in smooth muscle, it was recently demonstrated that AICAR-induced activation of AMPK could promote contractile differentiation of human coronary artery smooth muscle cells [30]. In accordance with that study, we found that AMPK activation promotes the contractile phenotype of smooth muscle cells in the unloaded portal vein to a similar extent as mechanical stretch.

In conclusion, our results demonstrate that stretch activates the AMPK signaling pathway and that stretch-sensitive miRNAs such as miR-144/451 can be involved in fine-tuning or long term control of this response. Furthermore, we show that AMPK activation, similar to stretch, can promote smooth muscle differentiation in both portal vein and aorta. AMPK activation may thus play a role in stretch-dependent contractile differentiation of vascular smooth muscle.

## Supporting Information

Figure S1 **Expression of miR-144 (A) and miR-451 (B) in smooth muscle cells of the intact aorta compared to cultured cells in passage 2 (p2) and passage 4 (p4); (ud = undetectable).**
(TIF)
